# Supplementary figures and images for: Water absorption through salivary gland type I acini in the blacklegged tick, Ixodes scapularis
Source: PeerJ. 2017 Oct 31;5:e3984. doi: 10.7717/peerj.3984 (PMC5669254; doi:10.7717/peerj.3984)

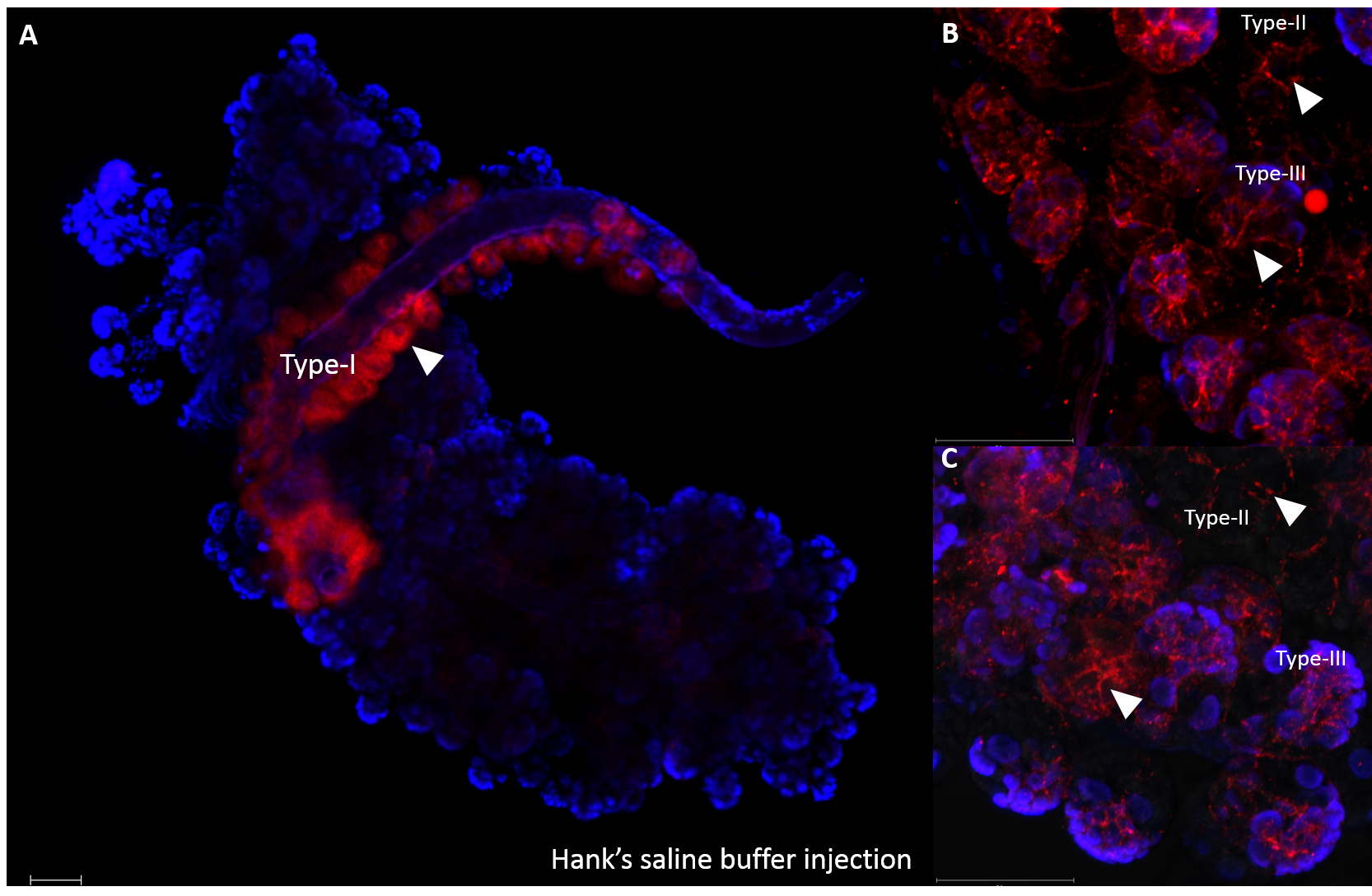

Supplement: Figure S1 — Red indicated positive immunoreactivities of Na/K-ATPase. Overview image of salivary glands (A). Close image focusing on type II and III acini (B & C). Scale bar indicated 50 um. [file peerj-05-3984-s002.jpg]

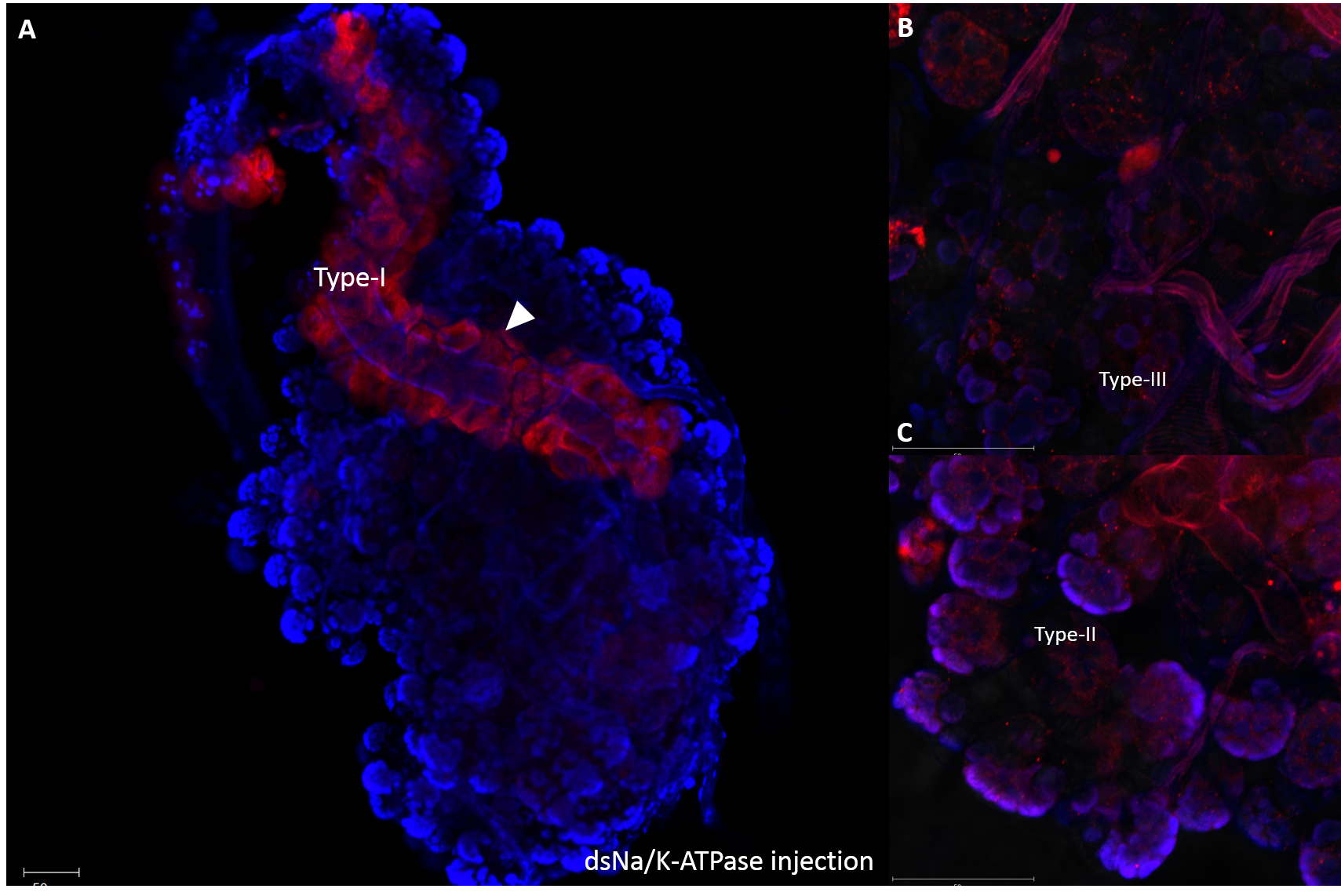

Supplement: Figure S2 — Arrow heads indicated immunoreactivities of Na/K-ATPase. Red indicated positive immunoreactivities of Na/K-ATPase. Overview image of salivary glands (A). Close image focusing on type II and III acini (B & C). Scale bar indicated 50 um. [file peerj-05-3984-s003.jpg]

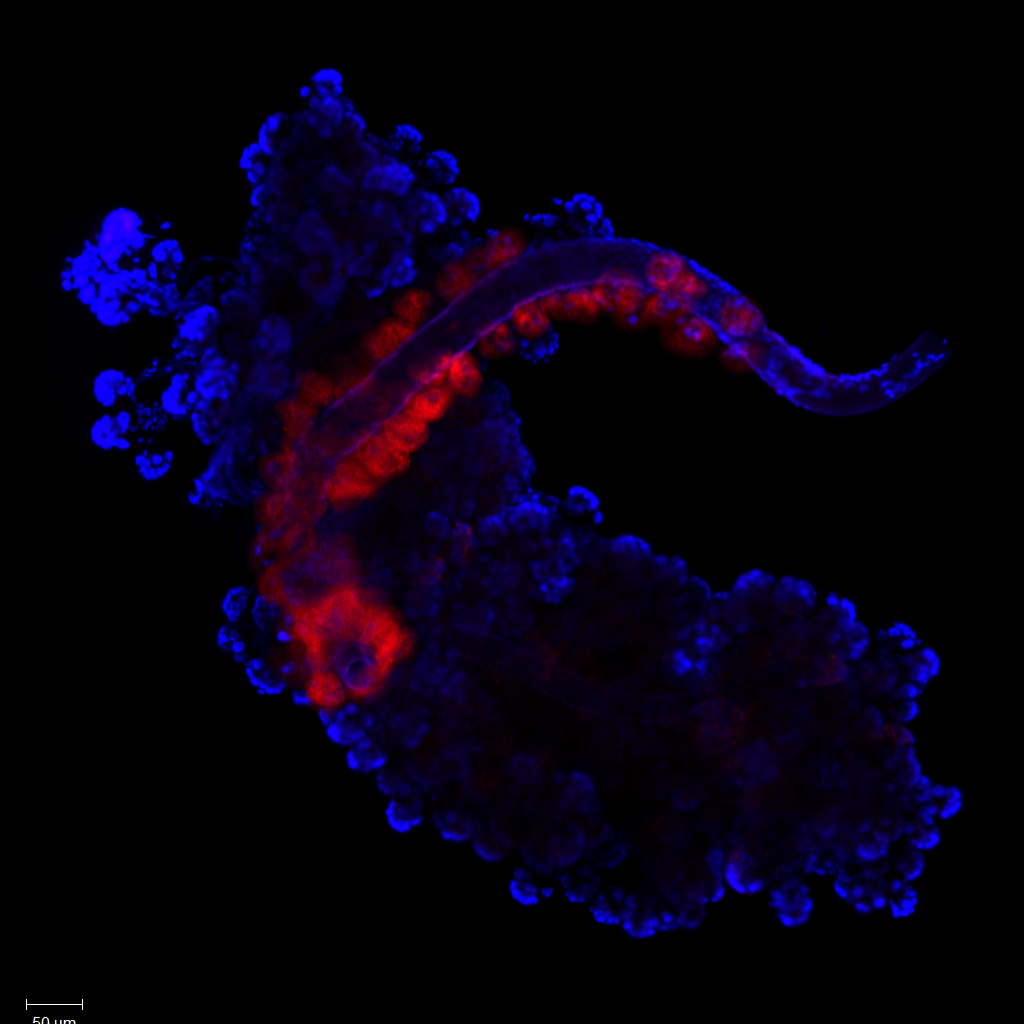

Supplement: Supplemental Information 2 [file peerj-05-3984-s005.jpg]

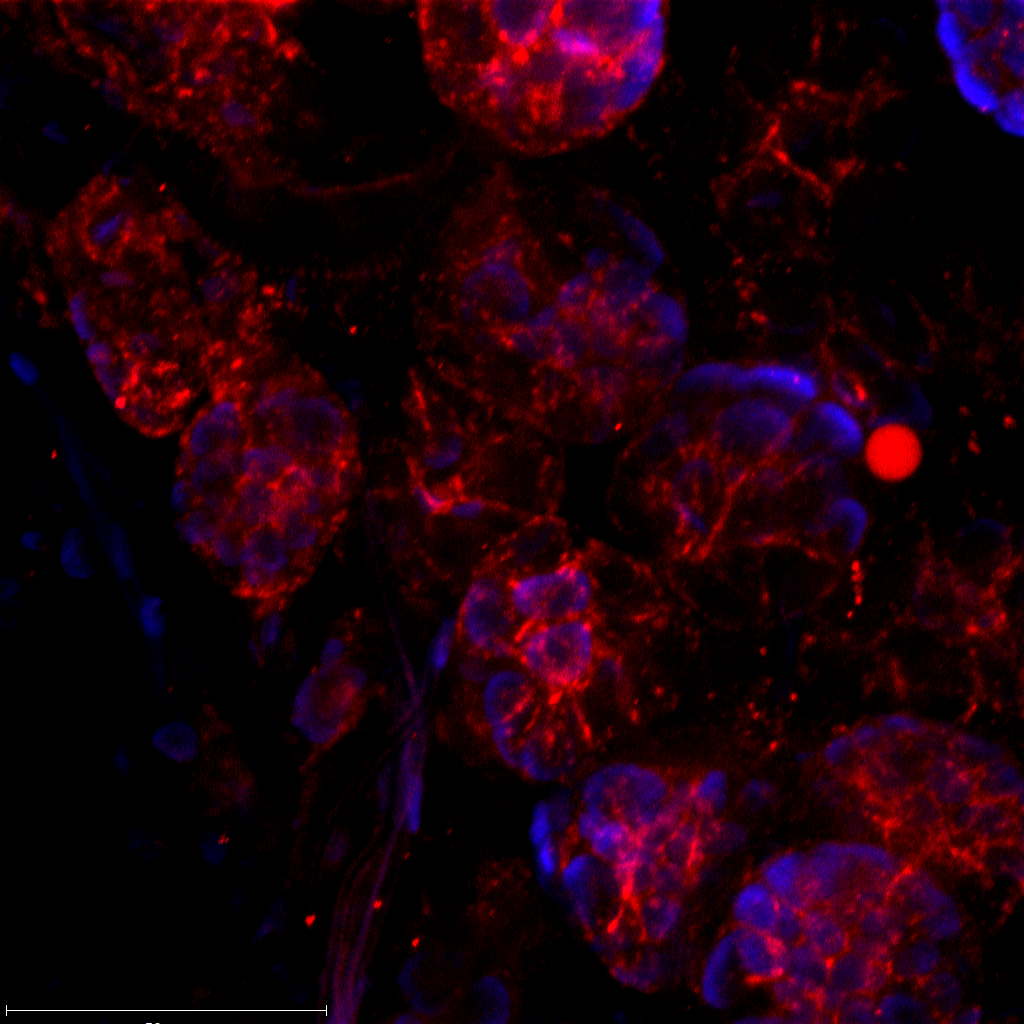

Supplement: Supplemental Information 3 [file peerj-05-3984-s006.jpg]

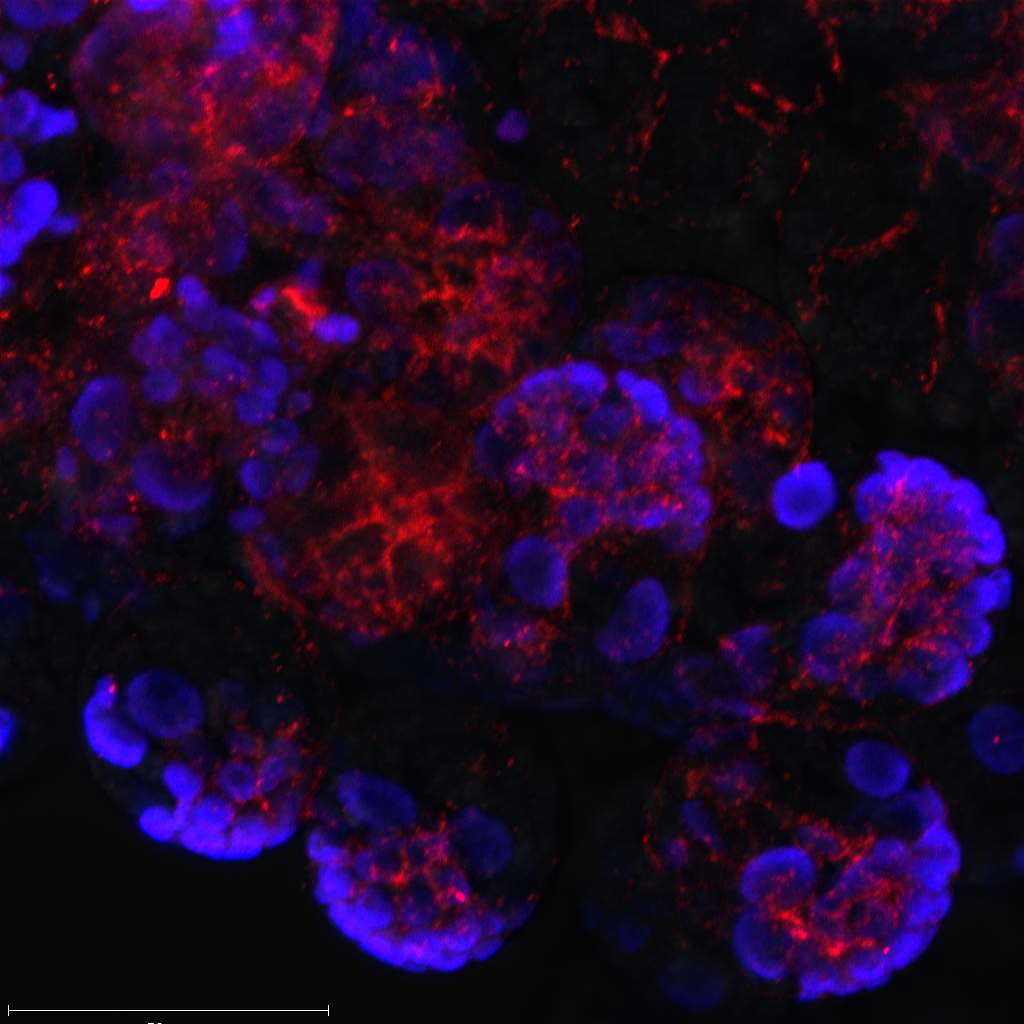

Supplement: Supplemental Information 4 [file peerj-05-3984-s007.jpg]

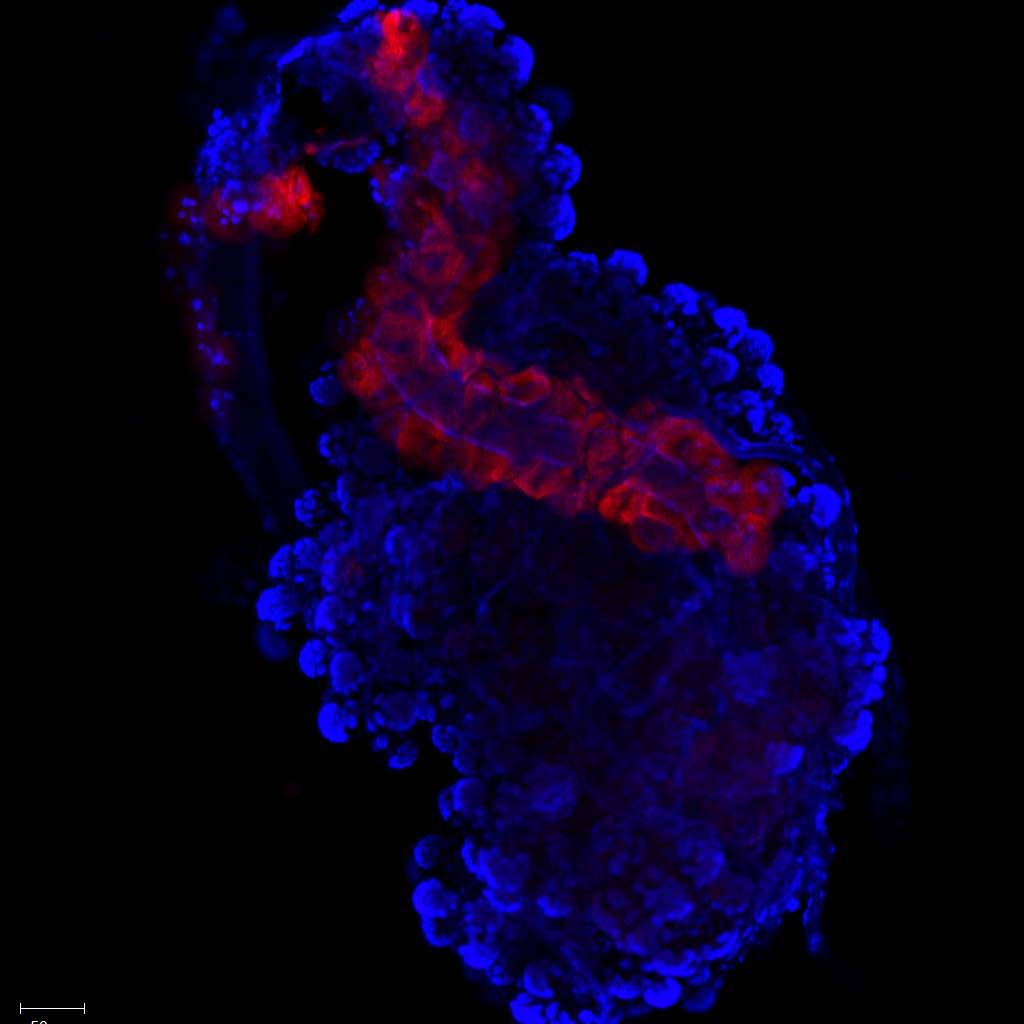

Supplement: Supplemental Information 5 [file peerj-05-3984-s008.jpg]

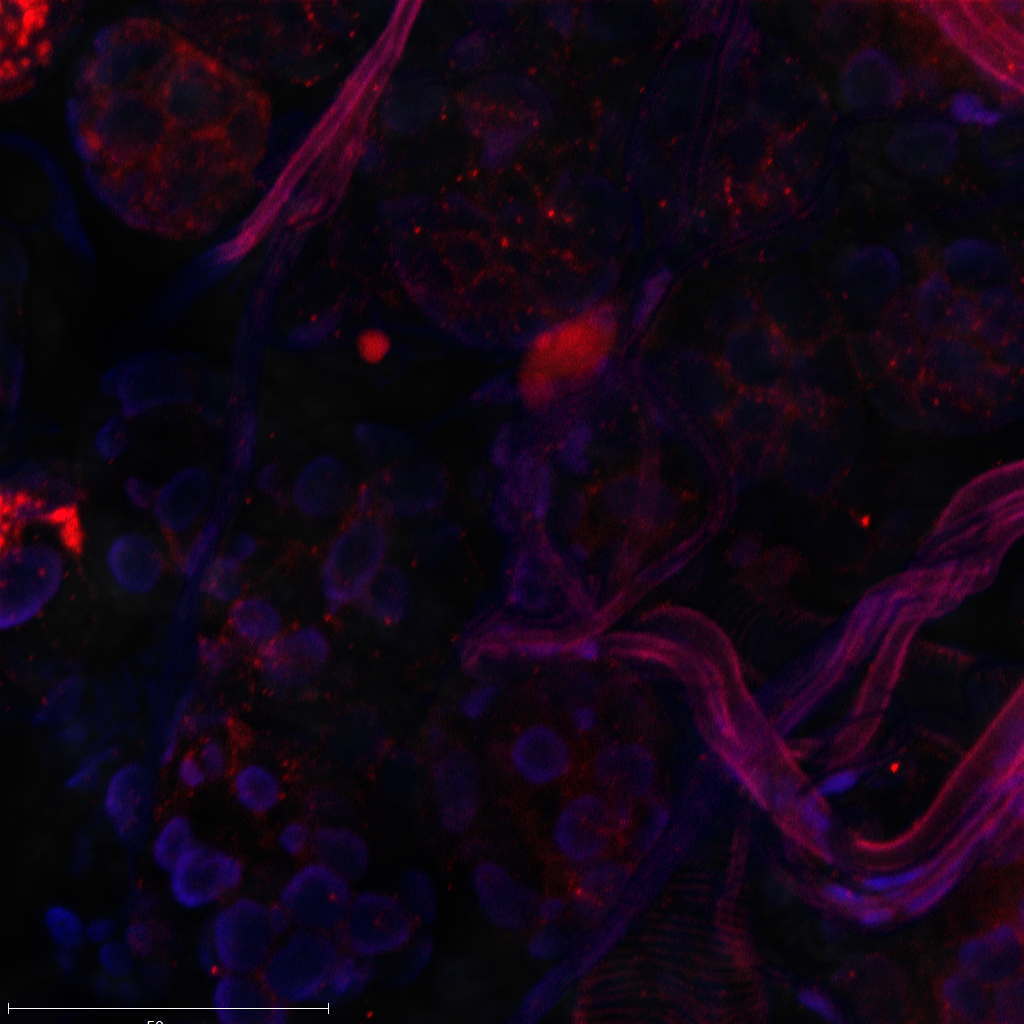

Supplement: Supplemental Information 6 [file peerj-05-3984-s009.jpg]

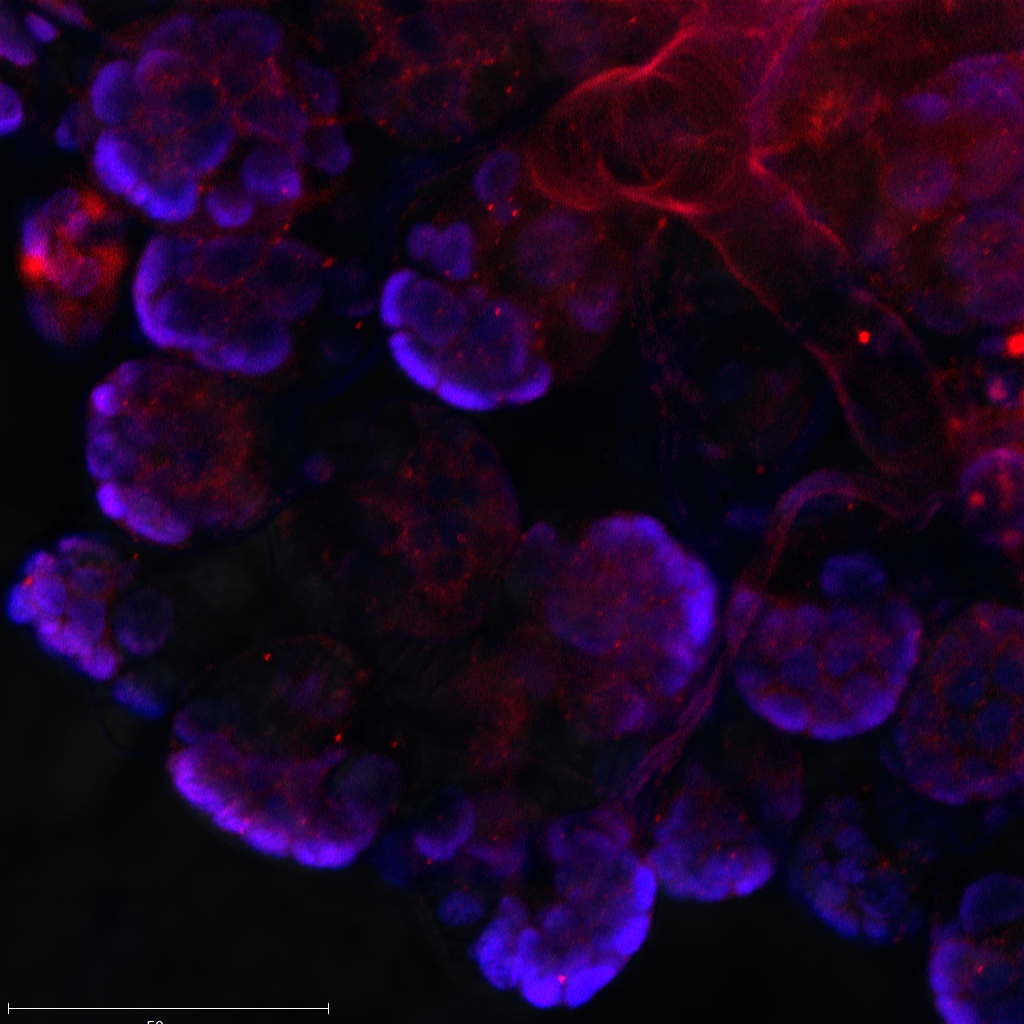

Supplement: Supplemental Information 7 [file peerj-05-3984-s010.jpg]
